# *Bacillus amyloliquefaciens* Confers Tolerance to Various Abiotic Stresses and Modulates Plant Response to Phytohormones through Osmoprotection and Gene Expression Regulation in Rice

**DOI:** 10.3389/fpls.2017.01510

**Published:** 2017-08-29

**Authors:** Shalini Tiwari, Vivek Prasad, Puneet S. Chauhan, Charu Lata

**Affiliations:** ^1^Council of Scientific & Industrial Research–National Botanical Research Institute Lucknow, India; ^2^Department of Botany, University of Lucknow Lucknow, India

**Keywords:** abiotic stress, cross-talk, expression, osmolytes, phytohormones, rhizobacteria

## Abstract

Being sessile in nature, plants have to withstand various adverse environmental stress conditions including both biotic and abiotic stresses. Comparatively, abiotic stresses such as drought, salinity, high temperature, and cold pose major threat to agriculture by negatively impacting plant growth and yield worldwide. Rice is one of the most widely consumed staple cereals across the globe, the production and productivity of which is also severely affected by different abiotic stresses. Therefore, several crop improvement programs are directed toward developing stress tolerant rice cultivars either through marker assisted breeding or transgenic technology. Alternatively, some known rhizospheric competent bacteria are also known to improve plant growth during abiotic stresses. A plant growth promoting rhizobacteria (PGPR), *Bacillus amyloliquefaciens* NBRI-SN13 (SN13) was previously reported by our lab to confer salt stress tolerance to rice seedlings. However, the present study investigates the role of SN13 in ameliorating various abiotic stresses such as salt, drought, desiccation, heat, cold, and freezing on a popular rice cv. Saryu-52 under hydroponic growth conditions. Apart from this, seedlings were also exogenously supplied with abscisic acid (ABA), salicylic acid (SA), jasmonic acid (JA) and ethephon (ET) to study the role of SN13 in phytohormone-induced stress tolerance as well as its role in abiotic and biotic stress cross-talk. All abiotic stresses and phytohormone treatments significantly affected various physiological and biochemical parameters like membrane integrity and osmolyte accumulation. SN13 also positively modulated stress-responsive gene expressions under various abiotic stresses and phytohormone treatments suggesting its multifaceted role in cross-talk among stresses and phytohormones in response to PGPR. To the best of our knowledge, this is the first report on detailed analysis of plant growth promotion and stress alleviation by a PGPR in rice seedlings subjected to various abiotic stresses and phytohormone treatments for 0, 1, 3, 10, and 24 h.

## Introduction

Rice (*Oryza sativa* L.) is the second most important staple crop across the globe having high caloric value. Various abiotic stresses such as salt, drought, and extreme temperatures, and biotic stresses are major threats for agricultural production and productivity worldwide. In fact abiotic stresses are reported to adversely affect the yields of staple food crops by 70% (Kaur et al., 2008; Mantri et al., 2012). Crop responses to these environmental stresses are manifested at physiological, biochemical and molecular levels from the early stage of seed germination to maturity and senescence. Expression of several thousand genes is known to be altered during various individual and multiple abiotic stresses (Miller et al., 2010; Dahro et al., 2016). It has now been well documented that the genes expressed under various abiotic stresses not only help in improving cellular tolerance by maintaining osmotic homeostasis but also by regulating stress responsive gene expression (Lata et al., 2015; Tiwari et al., 2017).

Several components of abiotic stress signaling have also been found to be regulated through plant growth regulators (PGRs) or phytohormones (Großkinsky et al., 2016). These are signal molecules which are produced within plants at very low concentrations with the ability to regulate various biological processes both locally and distally. Phytohormones namely auxins (AUX), cytokinins (CK), gibberellins (GA), abscisic acid (ABA), brassinosteroids (BR), salicylic acid (SA), jasmonates (JA) and ethylene play important roles in the regulation of plant developmental processes and signaling networks as they are involved either directly or indirectly in a wide range of biotic and abiotic stress responses and tolerance (Khan et al., 2012; Asgher et al., 2015; Srivastava et al., 2016; Tiwari et al., 2016). Numerous studies have shown the positive effects of phytohormones on the growth and development of a variety of crop plants (Cabello-Conejo et al., 2014). However, several phytohormones are also reported to be involved in pathogen-induced defense pathways and hence, have also been extensively used as biotic stress elicitors (Kim et al., 2004; Zhao et al., 2010; Koramutla et al., 2014). Each of these phytohormones is involved in different biological processes, thus affecting the growth and development of plants in a unique way. The effects of phytohormones on the plant vary with applied concentration, environmental factors, and on the physiological status of the plant at the time of application (Cabello-Conejo et al., 2014). Key plant hormones such as ABA, SA, JA, and ET are well known for their regulatory response under stress. As for example, various ethylene response factors such as *SlERF5* and *AtERF6* were established as master regulators of salt and drought stress tolerance in tomato and *Arabidopsis*, respectively (Pan et al., 2012; Dubois et al., 2013). It has also been anticipated that phytohormone synthesis and signaling play central role in response and adaptation to adverse environmental conditions (Bari and Jones, 2009; Lata et al., 2011b; Nafisi et al., 2015).

Interestingly, various plant growth promoting rhizobacteria (PGPR) are known for production of phytohormones as AUX, CK, GA and inhibit the synthesis of ethylene (Park et al., 2017). PGPR are also reported to increase the plant growth by reducing susceptibility to various environmental stresses (Nautiyal et al., 2013; Tiwari et al., 2016). The use of these beneficial microorganisms considered as one of the most promising methods for safe crop-management practices. Many soil microorganisms like *Azospirillum, Agrobacterium, Pseudomonas* and *Bacillus* produce phytohormones and also known to modulate endogenous level of phytohormones in plants thereby modulating the overall plant’s hormonal balance and its response to stress (Glick et al., 2007; Kundan et al., 2015). Members of *Bacillus* genus are among the most naturally abundant PGPR in the soil. A *Bacillus amyloliquefaciens* strain (NBRI-SN13, referred to as SN13) has been isolated from alkaline soil of Banthara, Lucknow and its characterization for various plant growth promotional attributes and stress tolerance such as, auxin and ACC deaminase production, solubilisation of tri-calcium phosphate and proline accumulation under salt stress were carried out earlier in our laboratory (Nautiyal et al., 2013). This PGPR was also reported as a biocontrol agent for *Rhizoctonia solani* infection in rice (Srivastava et al., 2016). However, to the best of our knowledge the role of this PGPR strain in various abiotic stresses and PGRs simultaneously has not been studied till date. Further, numerous molecular studies on phytohormones and stress related genes in rice have suggested the role of various stress signaling and regulatory pathways to play key roles in the cross-talk between phytohormone and biotic/abiotic stresses for plant protection (Du et al., 2013; Srivastava et al., 2016). Therefore, the aim of this study was to investigate the temporal effects of SN13 inoculation on various biochemical and molecular parameters in rice under different short-term abiotic stresses and phytohormone treatments, and delineating the mechanism of a possible cross-talk among them.

## Materials and Methods

### Plant Material, Inoculation and Stress Treatments

The experiment was conducted in a plant growth chamber at CSIR-NBRI, Lucknow, India with temperature oscillating between 25 ± 2°C (day) and 20 ± 2°C (night). A popular rice cultivar Saryu-52 was used for this study. The experiment was designed with two parameters control and 1% SN13 inoculated seedlings. Seeds of rice were surface sterilized with 0.1% HgCl_2_, transplanted and grown in hydroponics for 1 week in Hewitt medium. After 24 h of SN13 inoculation, both inoculated and uninoculated seedlings were subjected to various stresses. For salt and osmotic/drought stresses, 100 mM NaCl and 20% polyethylene glycol (PEG) was supplied, respectively (Srivastava et al., 2012; Nautiyal et al., 2013). For desiccation, seedlings were transferred to sterile Whatman filter paper and for heat, cold and freeze stresses, rice seedlings were transferred to 45°C, ∼4°C, and ≤0°C, respectively (Mishra et al., 2015). For various phytohormone treatments, both inoculated and uninoculated seedlings 100 μm each of ABA, SA, JA, and ethephon was supplied to the Hewitt medium as described elsewhere (Van Bockhaven et al., 2015). After respective treatments the root and shoot samples were harvested at 1, 3, 10, and 24 h for further studies. Unstressed seedlings (both inoculated and uninoculated) were maintained as control. All biochemical studies were performed with whole seedlings on the day of harvesting. Samples for qRT-PCR analyses were snap frozen in liquid nitrogen and stored at -80°C until further use. All experimental data are means of at least four independent biological replicates, while three biological as well three technical replicates were used for qRT-PCR analyses.

### Proline

Proline content was analyzed using ethanolic extract prepared by homogenizing ∼100 mg fresh tissue in 1 ml of 70% ethanol (Carillo and Gibbon, 2011; Tiwari et al., 2016). The reaction mixture constituted 1% w/v ninhydrin in 60% v/v acetic acid and 20% v/v ethanol, mixed with ethanolic extract in the ratio of 2:1. The 100 μl reaction mixture was then incubated in a water bath at 95°C for 20 min, cooled to room temperature, and absorbance was recorded at 520 nm in a microplate reader (Spectrum max plus; Molecular devices, Sunnyvale, CA, United States).

### Total Soluble Sugar

Total soluble sugar (TSS) content of rice seedlings was determined according to DuBois et al. (1956) with some modifications. About 100 mg of sample was homogenized in 3 ml of 80% methanol and was incubated at 70°C for 30 min. After incubation, equal volume (500 μl) of extract and 5% phenol each was mixed with 1.5 ml of 95% H_2_SO_4_ and further incubated in dark for 15–20 min. Absorbance was then measured in spectrophotometer (Spectrum max plus; Molecular devices, Sunnyvale, CA, United States) at 490 nm wavelength.

### Lipid Peroxidation

The level of lipid peroxidation (LP) in control and treated tissues was determined by measuring malondialdehyde (MDA) content via 2-thiobarbituric acid (TBA) reaction using modified protocol described by Heath and Packer (1968). About 100 mg of leaf tissues were homogenized in 500 μl of 0.1% (w/v) TCA and centrifuged for 10 min at 13,000 *g* at 4°C. Further 500 μl of supernatant was then mixed with 1.5 ml 0.5% TBA and incubated in water bath at 95°C for 25 min. Mixture was then incubated on ice for 5 min for termination of reaction. Absorbance of mixture was measured at 532 and 600 nm in a microplate reader (Spectrum max plus; Molecular devices, Sunnyvale, CA, United States).

### Quantitative Real Time (qRT) PCR Analysis of Stress-Responsive Genes from Rice

Total RNA was isolated from 1-week-old rice seedlings subjected to different durations of various abiotic stresses and phytohormones treatments with or without SN13-inoculation, using Tri-Reagent (Sigma, United States). DNase treatment was done using TURBO DNase (Ambion, United States) to remove DNA contamination from total RNA samples. The first strand of cDNA was synthesized using 1 μg of DNase free total RNA primed with oligo dT primers in a 20 μl reaction mix using Maxima H Minus M-MuLV reverse transcriptase (Thermo Scientific, United States) following manufacturer’s instructions. Before using as a template in quantitative real time-polymerase chain reactions (qRT-PCR), the cDNA products were five fold diluted with deionized water. qRT-PCR was performed using 2X Brilliant III SYBR^®^ Green QPCR (Agilent Technologies, United States) on Stratagene Mx3000P (Agilent Technologies, United States) in triplicates. A constitutive gene actin from rice was used as an internal control (Verma et al., 2016). The amount of transcript accumulated for each target gene normalized to the internal control was examined using 2^-ΔΔC_t_^ method (Livak and Schmittgen, 2001). The primers used for qRT-PCR analysis were designed from sequences of the respective genes downloaded from the National Center for Biotechnology Information (NCBI) using the IDT Primer Quest software (**Table 1**). The qRT-PCR cycling conditions were: initial denaturation at 95°C for 10 min, 95°C for 30 s, and 60°C for 1 min for 40 cycles followed by melt curve analysis at 95°C for 1 min, 60°C for 30 s, and 95°C for 30 s. The heat map for gene expression profiles were generated using TIGR MultiExperiment viewer (MeV 4) software package (Saeed et al., 2003).

**Table 1:** List of primers used in qRT-PCR analysis.

Gene(s)	NCBI accession no.	Primer sequence	Amplicon length
*DHN*	XM_015762124	F-5′ CTCCAGCTCATCCTCTGA 3′	79
		R-5′ GAGCTTCTCCTTGATCTTCTC 3′
*GST*	XM_015759043	F-5′ CCTACGTCACCAGAGTGAA 3′	81
		R-5′ TCTTGTTGCGGAGATCCT 3′
*LEA*	XM_015782086	F-5′ TGAGCAGGTGAAGAGCA 3′	111
		R-5′ GCAGAGGTGTCCTTGTTG 3′
*NAM*	XM_015760382	F-5′ GGAGTCCATGGAATTGGAAG 3′	101
		R-5′ GGCATGCCAAGAGGATTT 3′
*GRAM*	XM_015772803	F-5′ GCCCGCCTCCAAGAATACA 3′	69
		R-5′ TCTCCAAACCTCTTCCCCATCT 3′
*NRAMP6*	XM_015792141	F-5′ CTACTTCCTCAGCACAAAGC 3′	138
		R-5′ TTCCTGAACGTCAGGTAGAT 3′
*Actin*	XM_015761709	F-5′ GAGTATGATGAGTCGGGTCCAG 3′	143
		R-5′ ACACCAACAATCCCAAACAGAG 3′

### Statistical Analysis

All experimental results are expressed as mean with standard deviation (mean ± SD). To test the significance between mean values of control and stressed plants or SN13-inoculated unstressed and stressed plants, one way analysis of variance (ANOVA) was performed, and comparison among means was carried out using Duncan multiple range test (DMRT) at *P* < 0.05 with the help of SPSS software version 16.0 (SPSS Inc./IBM Corp., Chicago, IL, United States). All results were graphically presented using Graph Pad Prism software (version 5.03, San Diego, CA, United States). Principal component analysis (PCA) to delineate biochemical traits and gene expression differentiation among the treatments was performed using R 3.4.1 package.

## Results

### Modulation in Biochemical Parameters under SN13 Inoculation

To combat the adverse effects of various environmental stresses, plants have evolved complex mechanisms for their better survival, growth and adaptation. PGPR also regulate morpho-physiological, biochemical and molecular responses in plants. Therefore, in order to analyze the effects of SN13 on various biochemical parameters, rice seedlings were collected at different time points of various abiotic stresses namely, salt, drought, desiccation, heat, cold, and freeze, and phytohormone treatments viz. ABA, SA, JA, and ethephon after 1, 3, 10, and 24 h with or without SN13-inoculation.

#### Proline

**FIGURE 1:**
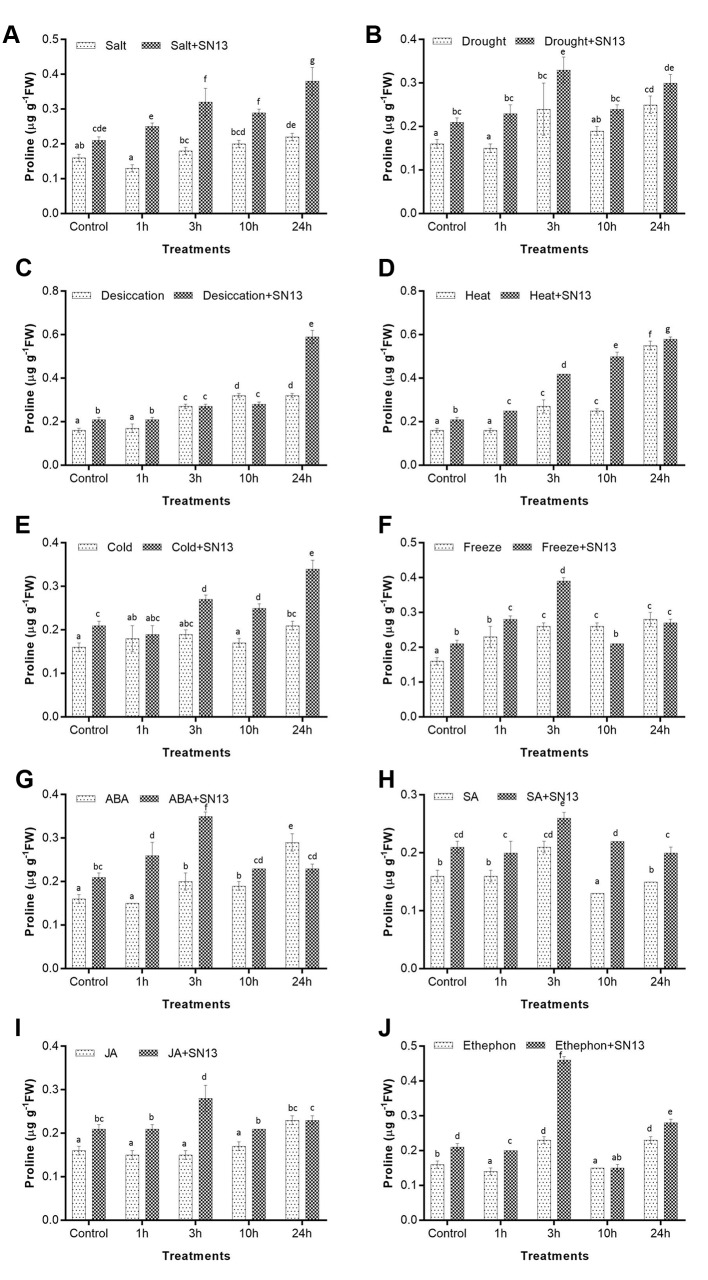
Determination of proline in rice exposed to salt **(A)**, drought **(B)**, desiccation **(C)**, heat **(D)**, cold **(E)**, freeze **(F)**, ABA **(G)**, SA **(H)**, JA **(I)**, and ethephon **(J)** stress at 1, 3, 10, and 24 h in the presence or absence of SN13. Data represent the means ± SD of four independent experiments. Different letters on the graph indicate significant differences according to Duncan’s test (*P* ≤ 0.05).

Proline is an osmoprotectant and has been suggested to contribute in osmoregulation during stress tolerance in plants. In inoculated and uninoculated rice seedlings, accumulation of proline was determined under all stress treatments. Interestingly, in all abiotic stresses and phytohormone treatments proline content was found to be significantly higher in SN13-inoculated seedlings in comparison to uninoculated. Salt, drought, desiccation, heat, and cold stress treatments led to significantly progressive increase in proline content at all stress durations in inoculated seedlings than uninoculated ones with maximum accumulation at 3 h in most of the stresses (**Figures 1A–J**). In comparison to control, uninoculated seedlings at 24 h under salt, drought, desiccation, heat, and cold showed percentage increment of 37, 56, 100, 243, and 31% while SN13-inoculated 24 h stressed seedlings showed increase in proline content by 81, 43, 180, 176, and 62%, respectively (**Figures 1A–E**). On the other hand, inoculated rice seedlings subjected to salt, drought, desiccation, and cold stresses showed enhanced proline content by 72, 20, 84, and 62%, respectively, at 24 h in comparison to the respective stressed uninoculated seedlings (**Figures 1A–C,E**). However, at 24 h of heat stress only 5% increase in proline content was recorded in inoculated seedlings as compared to uninoculated (**Figure 1D**). Furthermore, SN13 inoculated seedlings under freeze and all phytohormone treatments namely, ABA, SA, JA, and ethephon showed comparatively higher proline content viz. 86, 66, 24, 33, and 119%, respectively, at 3 h of stress as compared to inoculated controls (**Figures 1F–J**). While at 24 h of abovementioned stresses, a decline in proline content by 30, 34, 23, 22, and 39%, respectively, was observed in inoculated seedlings. However, at the same time point, a less significant difference in uninoculated rice seedlings was recorded for proline content under freeze and phytohormone treatments.

#### Total Soluble Sugar

**FIGURE 2:**
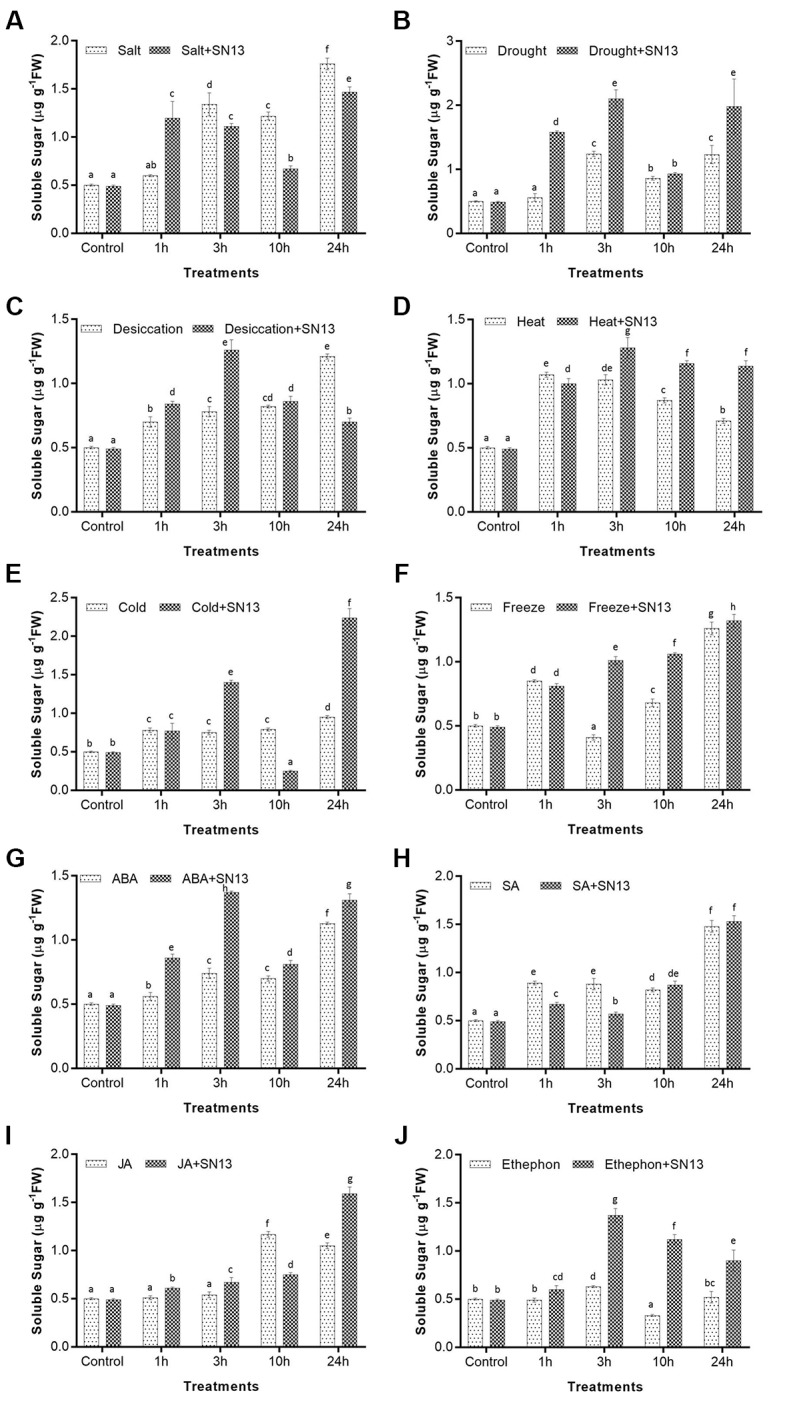
Determination of total soluble sugar in rice exposed to salt **(A)**, drought **(B)**, desiccation **(C)**, heat **(D)**, cold **(E)**, freeze **(F)**, ABA **(G)**, SA **(H)**, JA **(I)**, and ethephon **(J)** stress at 1, 3, 10, and 24 h in the presence or absence of SN13. Data represent the means ± SD of four independent experiments. Different letters on the graph indicate significant differences according to Duncan’s test (*P* ≤ 0.05).

Total soluble sugar is also a well-known compatible osmolyte that help plants to withstand various environmental stresses via maintaining the stability of membranes. Accumulation of soluble sugar was significantly higher in SN13-inoculated plants in comparison to the uninoculated seedlings at all durations of all applied stresses. Both inoculated and uninoculated seedlings during salt, drought, desiccation, heat, and cold stress showed significantly progressive increment in TSS content till 3 h and at 24 h of stress treatments (**Figures 2A–E**). In comparison to inoculated unstressed seedlings, the inoculated seedlings subjected to abovementioned stresses, showed enhancement in TSS content by 126, 328, 157, 161, and 185%, respectively, at 3 h while an increment between ∼200 and 400% was recorded for each stress at 24 h in inoculated stressed seedlings. Similarly, maximum TSS content (∼180%) was found at 3 h of ABA and ethephon treatments in inoculated seedlings as compared to the inoculated control while after a decline at 10 h, an increment by 167 and 84%, respectively, was observed at 24 h (**Figures 2G,J**). Uninoculated seedlings under salt, drought, desiccation and heat stresses followed similar pattern as inoculated seedlings at all time points of stress treatments but degree of accumulation of TSS was comparatively lower than inoculated seedlings. Furthermore, both inoculated and uninoculated seedlings under freeze, SA and JA treatments showed progressive increase in level of soluble sugar with increasing duration. Under these treatments maximum level of soluble sugar was recorded at 24 h (∼170, ∼210, and ∼200%, respectively) in both inoculated and uninoculated rice seedlings, as compared to their respective controls (**Figures 2F,H,I**).

#### Lipid Peroxidation

**FIGURE 3:**
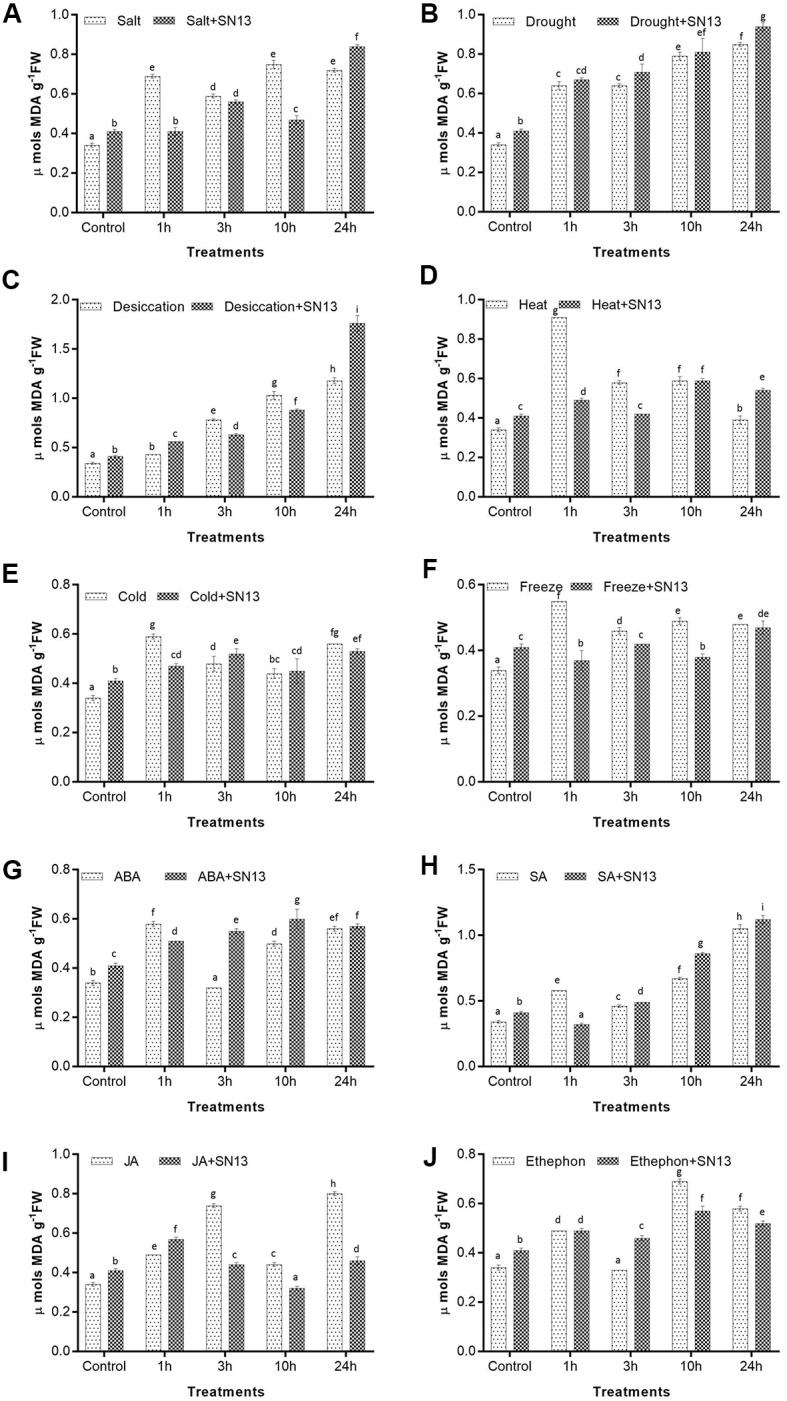
Determination of MDA content in rice exposed to salt **(A)**, drought **(B)**, desiccation **(C)**, heat **(D)**, cold **(E)**, freeze **(F)**, ABA **(G)**, SA **(H)**, JA **(I)**, and ethephon **(J)** stress at 1, 3, 10, and 24 h in the presence or absence of SN13. Data represent the means ± SD of four independent experiments. Different letters on the graph indicate significant differences according to Duncan’s test (*P* ≤ 0.05).

To check the membrane integrity lipid peroxidation analysis was performed by measuring total MDA content. During stress, MDA works as an indicator of extent of lipid peroxidation in living tissues, i.e., an increase in MDA denotes more membrane damage and vice versa. At all given treatments membrane destruction significantly increased with stress progression (**Figures 3A–J**). Under controlled conditions, MDA content was found to be slightly higher in inoculated seedlings as compared to the uninoculated seedlings, while under salt, drought, desiccation, heat, cold, and freeze stresses, SN13 inoculated seedlings showed significantly equivalent or lower MDA content in comparison to the uninoculated seedlings till 10 h (**Figures 3A–F**). However, at 24 h of salt, drought, desiccation, and heat stress, SN13-inoculated seedlings showed slightly higher MDA content by 17, 11, 48, and 38%, respectively, in comparison to the uninoculated seedlings, while the same was not observed under cold and freezing stresses where the MDA content were lower by ∼5% than uninoculated seedlings. On the other hand, the inoculated and uninoculated rice seedlings treated with ABA and ethephon at 3 h showed improved membrane integrity on account of lower MDA content, i.e., by 3 and 12% in ABA and 6 and 34% in ethephon, respectively, while an increase in MDA accumulation was observed till 24 h in both (**Figures 3G,J**). At the same time both inoculated and uninoculated JA-treated seedlings, showed irregular pattern for membrane ion leakage as MDA content was found to increase till 3 h (∼10 and 117%, respectively) and at 24 h (∼12 and ∼117%, respectively) (**Figure 3I**). Both inoculated and uninoculated seedlings under desiccation and SA treatment showed gradual increase in the level of MDA content until 10 h with an abrupt increase by ∼200% at 24 h (**Figures 3C,F**).

### SN13 Inoculation Alters Stress-Responsive Gene Expression

**FIGURE 4:**
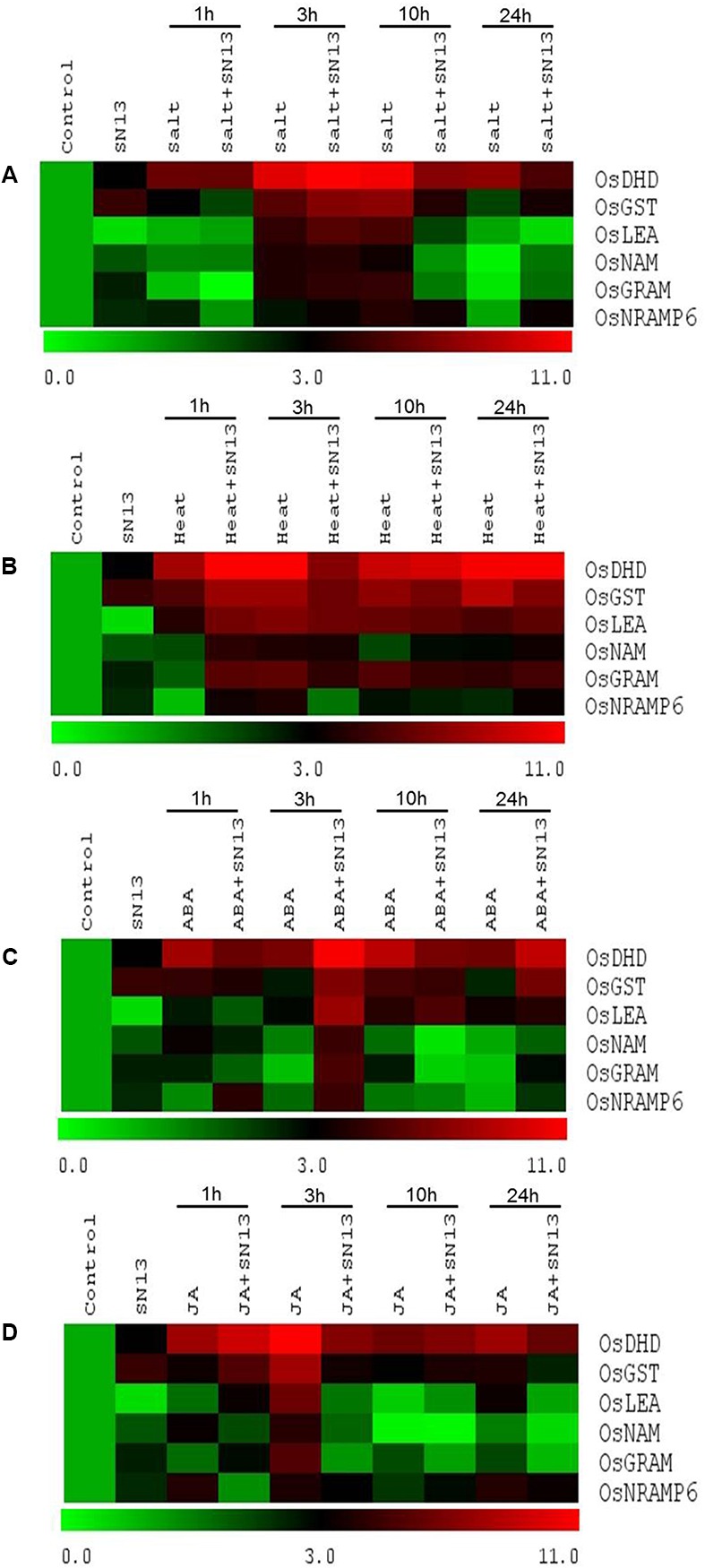
Differential expression of genes in rice exposed to salt **(A)**, heat **(B)**, ABA **(C)**, and JA **(D)** stress at 1, 3, 10, and 24 h in the presence or absence of SN13. The heat map has been generated based on the fold-change values in the treated sample when compared with its unstressed control sample. The color scale for fold-change values is shown at the bottom.

In order to verify our results for biochemical analyses, qRT-PCR analysis of six stress-responsive genes namely, dehydrin (*DHN*), glutathione S-transferase (*GST*), late embryogenesis abundant (*LEA*), no apical meristem (*NAM*), glucosyltransferases, Rab-like GTPase activators, myotubularin (*GRAM*) and natural resistance-associated macrophage protein 6 (*NRAMP6*) genes were carried out at all-time points in rice roots under two representative abiotic stresses, i.e., salt and heat, and two representative phytohormone treatments, i.e., ABA and JA. All genes showed significant differential expression under abovementioned treatments and the gene expression profiling data was also found to be considerably in correlation with our biochemical results under these four stresses. Under unstressed conditions, the inoculated seedlings showed ∼3-fold upregulation in *DHN* expression than uninoculated ones (**Figures 4A–D**). In both inoculated and uninoculated seedlings expression of *DHN* in salt stressed plants increased gradually till 3 h with a ∼11-fold induction as compared to control. In case of ABA treated inoculated seedlings maximum expression of *DHN* was observed at 3 h (∼11-fold) followed by 24 h (∼9-fold) as compared to control (**Figure 4C**). In contrast, uninoculated seedlings showed upto ∼9-fold increase in *DHN* expression after 1 and 10 h of ABA treatment. Heat and JA stressed seedlings showed somewhat similar pattern of *DHN* expression (**Figures 4B,D**). Under heat and JA treatments, uninoculated seedlings showed maximum *DHN* expression (∼12-fold) at 3 h whereas SN13-inoculated seedlings showed maximum upregulation of ∼12- and ∼9-fold, respectively, at 1 h for both stresses.

Under unstressed conditions, *GST* expression was found to be upregulated by ∼4.5-fold in inoculated seedlings as compared to uninoculated (**Figures 4A–D**). As compared to control, *GST* expression was found to be highest (∼7-fold) in inoculated seedlings at 3 h and in uninoculated seedlings at 10 h of salt stress. Further, 1 h heat stressed inoculated seedlings showed highest *GST* expression (∼8-fold) while a ∼9-fold expression was observed at 24 h in uninoculated rice seedlings (**Figure 4B**). In case of ABA treatment, ∼7-fold increase was observed in inoculated seedlings at 3 and 24 h as compared to control while uninoculated seedlings showed ∼5-fold of upregulation at 10 h (**Figure 4C**). JA treated seedlings with SN13- inoculation showed ∼6-fold upregulation in *GST* expression at 1 h while uninoculated seedlings showed ∼8-fold expression at 3 h as compared to control (**Figure 4D**). However, a gradual decline in *GST* expression was recorded till 24 h in JA-treated seedlings under both inoculated and uninoculated seedlings.

On the other hand, SN13-inoculated control seedlings showed upto two fold upregulation in *NAM* and *GRAM* gene expression while no significant difference was found in the expression of *LEA* gene as compared to uninoculated control (**Figures 4A–D**). Under salt stress, *LEA*, *NAM* and *GRAM* genes showed highest expression (∼4- to ∼5.5-fold) in SN13-inoculated seedlings at 3 and 10 h of stress as compared to control (**Figure 4A**). ABA-treated seedlings showed similar expression patterns for *LEA*, *NAM*, and *GRAM* genes as under salt stress (**Figure 4C**). Inoculated seedlings showed highest expression of *LEA*, *NAM*, and *GRAM*, i.e., ∼7.8-, ∼4.7- and ∼5.3-fold, respectively, after 3 h of ABA application, while a gradual decline was observed in both inoculated and uninoculated seedlings till 24 h of stress. However, at 3 h of JA treatment, uninoculated seedlings showed upto six fold increment in *LEA*, *NAM*, and *GRAM* expression (**Figure 4D**). Furthermore, no significant change in expression of these genes was recorded till 24 h of stress. Unlike other stresses, all three genes showed distinct expression pattern under heat stress (**Figure 4B**). Maximum expression of *NAM* and *GRAM* genes was recorded to be ∼4- and ∼5-fold, respectively, in both inoculated and uninoculated seedlings at 3 h of heat stress, thereafter a slight reduction was observed in their expression with stress progression till 24 h time point. Maximum expression of *LEA* (∼5-fold) was recorded at 1 h of heat stress as compared to control. Interestingly, its expression was found to be more or less maintained till 24 h in both inoculated and uninoculated seedlings (**Figure 4B**).

Expression of *NRAMP6* gene was ∼2-fold higher in inoculated seedlings as compared to uninoculated seedlings under unstressed condition (**Figures 4A–D**). Under salt stress *NRAMP6* expression was highest at 10 h in both uninoculated and inoculated seedlings (∼4- and ∼3.5-fold, respectively) in comparison to control (**Figure 4A**). After 10 h of salt stress, a ∼3-fold reduction in *NRAMP6* expression was observed in uninoculated seedlings while no significant difference was found in inoculated seedlings. Expression of *NRAMP6* under heat stress was highest at 1 h in inoculated seedlings and at 3 h in uninoculated seedlings (∼4-fold) in comparison to control (**Figure 4B**). A gradual decline in *NRAMP6* expression was observed in uninoculated seedlings while inoculated seedlings showed reduction only at 3 h of heat stress thereafter its expression increased upto ∼3-fold at 24 h. In ABA-treated uninoculated seedlings, a ∼1.5-fold expression of *NRAMP6* was observed at all-time points while SN13-inoculated seedlings showed ∼4-fold upregulation after 1 and 3 h of ABA treatment with a significant reduction (∼2-fold) at 10 and 24 h (**Figure 4C**). On the other hand, uninoculated JA-treated seedlings showed ∼4-fold *NRAMP6* expression at 1, 3, and 24 h and ∼2-fold at 10 h of stress as compared to control. While inoculated seedlings showed ∼3-fold up-regulation in *NRAMP6* expression at 3, 10, and 24 h in comparison to control (**Figure 4D**). Expression patterns of all six genes, i.e., *DHN*, *GST*, *LEA*, *NAM*, *GRAM* and *NRAMP6* under salt, heat, ABA and JA treatments in both inoculated and uninoculated seedlings at all-time points have also been provided as individual graphical representations in supplementary information (**Supplementary Figures S1A–F**).

### PCA of Abiotic Stresses and Phytohormone Treatments

**FIGURE 5:**
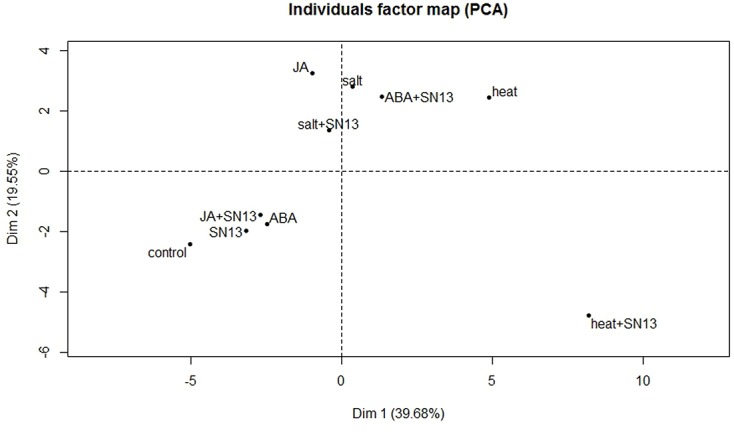
Principal component analysis biplot of biochemical traits and gene expression of rice under abiotic stresses and phytohormone treatments in the presence or absence of SN13.

In order to better understand the relationships, similarities and dissimilarities among the results for biochemical traits and gene expression, a multivariate PCA was carried out. The multivariate PCA lets a large number of variables to be lessened to only a few which largely account for majority of the variance in the observed experimental results. PCA was applied among biochemical parameters (proline, TSS and LP) and gene expression of all six genes to determine the interaction between abiotic stresses (salt and heat) and phytohormones (ABA and JA) under the influence of PGPR at all-time intervals (**Supplementary Figures S2A–D**). Dimension 1 (Dim1) and Dim2 accounted for ∼80% of the total variance at all four time intervals. More specifically after assembling all-time points, Dim1 accounted for 39.68%, and Dim2 was responsible for 19.55% of the total variance (**Figure 5**). Mainly two clusters were formed in the biplot. Cluster including salt, heat, JA, salt+SN13 and ABA+SN13 have positive values at both the axis while the cluster containing control, SN13, ABA and JA+SN13 lied at negative values of both the axis. Heat+SN13 is located far from the clusters indicating dissimilar response from other clusters. In accordance, distinct clusters formed in biplot clearly exhibited correlation between abiotic stresses and phytohormones under influence of SN13 in rice.

## Discussion

In the present scenario of global climate change, adverse environmental conditions lead to significant reduction in growth, development and yield of crop plants (Lata et al., 2015). The development of new crop varieties is one of the most established methods of crop improvement for stress management. Since, transgenic technology and molecular breeding are time consuming and labor intensive processes; use of plant growth promoting microbes is gaining wide popularity these days as an alternate strategy for improving stress tolerance of crop plants (Glick, 2014; Tiwari et al., 2016). Among various PGPR genus, *Bacillus* and *Pseudomonas* are most extensively studied rhizobacteria that promote plant growth and development (Kumar et al., 2011). Earlier studies have reported several strains of *Bacillus* spp. viz. *B. amyloliquefaciens*, *B. licheniformis*, *B. megaterium*, *B. pumilus*, and *B. subtilis*, etc., as well known rhizosphere residents of many crops with plant growth promoting activities (Kloepper et al., 2004; Kumar et al., 2011). Nautiyal et al. (2013) studied PGPR traits of *B. amyloliquefaciens* and its effects on rice during salt stress. Srivastava et al. (2016) also reported *B. amyloliquefaciens* mediated enhanced production of rice under *Rhizoctonia* infection. However, our study demonstrates the regulatory role of PGPR, *B. amyloliquefaciens* NBRISN13 under various abiotic stresses and phytohormone treatments through biochemical studies and gene expression analyses of six stress responsive genes in 1-week-old rice seedlings. Unfavorable environmental conditions during early seedling stage in rice result in drastic reduction in growth resulting in lower yield potential and poor grain quality (Manikavelu et al., 2006; Farooq et al., 2009). To the best of our knowledge, until now none of the studies have been conducted to evaluate the effects of various abiotic stresses and phytohormones on stress tolerance abilities of rice at early seedling stage in the presence of a PGPR.

Accumulation of compatible osmolytes such as proline, soluble sugars, glycine betaine, trehalose, etc., help plants to overcome abiotic stresses by maintaining osmotic turgor (Tiwari et al., 2016; Zandalinas et al., 2017). Elevated levels of proline, TSS, betaine in plants have also been correlated with enhanced stress tolerance in previous studies (Lata et al., 2015; Tiwari et al., 2016). Accordingly this study also reports an increase in proline and TSS content in rice cv. Saryu-52 subjected to six abiotic stresses and four phytohormone treatments. In general, proline and TSS content of inoculated rice plants showed significant time-dependent increase as compared to the non-inoculated seedlings under all stresses. This increase in the level of proline and TSS upon SN13 inoculation can be associated with improved plant health under various stresses resulting in better stress tolerance of rice seedlings. Similar increase in proline and TSS content have also been reported in salt stressed wheat seedlings inoculated with halo-tolerant PGPR *Dietzia natronolimnaea* (Bharti et al., 2016). Accordingly, Khan et al. (2016) observed that the inoculation with *Bacillus pumilus* improved proline content of rice seedlings subjected to salt stress. Nautiyal et al. (2013) also reported enhanced proline content in 1-month-old rice seedlings inoculated with SN13 when subjected to salt stress. Similar observations have also been reported in maize under drought stress (Kandowangko et al., 2009; García et al., 2017). Increased level of soluble sugar in maize on inoculation with *Pseudomonas* spp. under drought stress was correlated with better stress tolerance (Sandhya et al., 2010). Interestingly, high proline and TSS accumulation was observed after 3 h of stress progression in most of the abiotic stresses and all phytohormone treatments. This could be due to an osmotic adjustment as a result of increased synthesis of osmolytes. Similar observation was made by Jain and Chattopadhyay (2010) in chickpea subjected to drought stress. Further our results indicated a more or less similar pattern of proline accumulation under drought, salt, ABA, SA, JA, and ethephon treatments with a higher accumulation in inoculated seedlings as compared to uninoculated ones. Likewise, TSS accumulation was found to follow a similar pattern as that of proline accumulation in salt, drought, dessication, cold, ABA, and ethephon treatments indicating a complex SN13-mediated cross-talk among various abiotic stresses and phytohormones. Though such observations have not been reported earlier for any plant–PGPR interaction involving so many abiotic stresses and phytohormones, however, there are numerous reports on extensive cross-talk among various abiotic stresses and phytohormones (Fahad et al., 2015; Tiwari et al., 2017). Several phytohormones like ABA, SA, JA, and ET have been reported to be central to drought, salt, cold, and heat stress responses in various plants (Lata et al., 2011b) while other phytohormones such as gibberellins, brassinosteroids, auxin, cytokinins, etc., interact with other phytohormones and stress-related genes to maintain a balanced plant growth and development (Kohli et al., 2013).

Malondialdehyde (MDA) is one of the end products of polyunsaturated fatty acids peroxidation in phospholipids and is responsible for cell membrane damage (Sharma et al., 2012). MDA accumulation is an indication of stress-induced LP of cellular membrane lipids and is often considered a marker for increased oxidative damage (Lata et al., 2011a). In our findings LP was significantly lower at all durations of short-term salt, drought, and desiccation stresses upon SN13 inoculation except at late stress, i.e., 24 h. This might be possible that initially layer of mucilage around root protects plant by direct exposure to stress but later bacterial adherence to root or root cortex changes membrane permeability to some extent and leads to slight increase in membrane damage. Ongena and Jacques (2008) have also reported interaction of *Bacillus* with plant membrane as a biocontrol via altering membrane structure. Pandey et al. (2016) and Meena et al. (2017) reported increased MDA content in rice treated with *Trichoderma*. However, estimation of LP at subsequent later durations (beyond 24 h) of bacterial colonization may be an interesting subject area of study in these stresses. One of the previous studies also reported an increase in MDA content at initial stages of *Burkholderia phytofirmans* inoculation in *Vitis vinifera* under cold stress and a decrease in MDA content was recorded at later stages suggesting the stress ameliorating properties of the PGPR (Theocharis et al., 2012). On the other hand, heat, cold, and freeze stresses did not show significant alteration in MDA content upon SN13 inoculation at all durations. This may be due to the fact SN13 may not be directly involved in maintaining membrane integrity under these stresses owing to their poor tolerance levels to extreme temperatures.

Further, PGPR-mediated activation of numerous genes in response to abiotic stresses has recently been reported in many crop plants including rice (Nautiyal et al., 2013; Kim et al., 2014; Tiwari et al., 2016). However, molecular basis of PGPR–plant interactions with respect to abiotic stress tolerance and phytohormone treatments in rice remain largely unknown. Therefore, in order to understand the changes at molecular level during rice–SN13 interaction under various stresses, expression analyses of a few stress-responsive genes through qRT-PCR was performed.

*LEA* and *DHN* are mainly involved in stress tolerance and hence, act as marker genes for plant stress response (Tiwari et al., 2016). Overexpression of these genes has been reported to provide tolerance to various abiotic stresses in several crop plants (RoyChoudhury et al., 2007; Kumar et al., 2014). Further, overexpression of dehydration responsive element binding (*DREB*) genes in *Arabidopsis* and rice is also known to increase the expression of *LEA* and dehydrins (Lata and Prasad, 2011). In present study, the expression of these genes increase at all-time points with maximum expression at 3 and 10 h of all four applied treatments in comparison to control, indicating their correlation with an increased osmolyte synthesis at these durations. While SN13-inoculation relatively down regulates the expression of *LEA* and *DHN* under salt and heat stress as well as ABA treatment at all durations indicating the crucial role of SN13 in stress alleviation in 1-week-old rice seedlings. However, *LEA* and *DHN* expression is significantly higher at 3 h of the abovementioned stresses which may be due to their active role in osmolyte biosynthesis and subsequently osmotic adjustment. This result is also in accordance to our biochemical results for proline and TSS. Recently, *Trichoderma harzianum*, a rhizosphere occupants reported in stress mitigation in rice genotypes due to upregulation of dehydrin and other genes (Pandey et al., 2016; Meena et al., 2017). Similarly, the expression of *LEA* increases on application of *B. subtilis* in *Brachypodium* under drought stress (Gagne-Bourque et al., 2015) as well as in chickpea upon *P. putida* inoculation (Tiwari et al., 2016). Further, increased expression of *DHN* was reported by Kumar et al. (2014) in rice and Kosová et al. (2014) in barley and wheat under salt, drought and cold stresses. Interestingly, many *DHNs* have been identified in plants including *Arabidopsis thaliana* and wheat that are up-regulated by exogenous ABA under drought stress (Lv et al., 2017). Richard et al. (2000) reported exogenous application of JA leads to increase in *DHN* expression in white spruce. Differential expression of *DHN* and *LEA* under salt, heat, ABA, and JA treatments in our study suggests an extensive SN13- mediated cross-talk among them. *P. putida* treated tolerant and sensitive chickpea cultivars also showed differential gene expression for abiotic stress-responsive genes *DHN* and *LEA* as well as for *MYC2* and *PR1* genes which involved in JA and SA signaling, respectively, under drought stress (Tiwari et al., 2016).

Glutathione S-transferases (GSTs) are ubiquitous enzymes with antioxidant properties that help in detoxification via converting oxidatively produced compounds to reduced glutathione, thus facilitating their removal, sequestration, or metabolism (Dalton et al., 2009). Increased expression of GST in SN13-inoculated rice seedlings at all stress durations with maximum expression at 3 h indicates an induction of this defense enzyme due to SN13 colonization. Similar observation was also reported by Srivastava et al. (2012) in *Arabidopsis* upon *P. putida* inoculation. Kandasamy et al. (2009) also reported upregulation in GST expression on treatment of *P. fluorescens* in rice.

*NAM* TFs have been reported to play significant role in abiotic stress tolerance in various crop plants (Nakashima et al., 2009). Increased expression of *NAM* gene on exposure to various stresses in rice is in accordance to previous studies (Nguyen et al., 2015; Tiwari et al., 2016). Further, its relatively increased transcript accumulation in SN13-inoculated rice seedlings at all stress durations with a few exceptions shows a positive regulation of *NAM* by SN13. Wang et al. (2005) and Tiwari et al. (2016) have also demonstrated *Pseudomonas* spp.-induced expression of *NAM* in *Arabidopsis* and chickpea, respectively, via gene expression profiling studies, and their role were speculated in PGPR-mediated stress tolerance.

*GRAM* domain containing genes are likely to be involved in membrane associated processes such as intracellular protein or lipid binding signaling pathways (Doerks et al., 2000; Jiang et al., 2008). Baron et al. (2014) also reported that *GRAM*-domain containing genes show responsiveness to several phytohormone and abiotic stresses. Upregulation of *GRAM* by ABA was also reported (Liu et al., 1999; Jiang et al., 2008). In our findings, increased expression of *GRAM* in both inoculated and uninoculated seedlings at early stages of stress treatments suggest an SN13-mediated gene expression modulation during initial stress signal transduction events in rice.

*NRAMP* genes in plants are known to encode intracellular metal transporters with capacity to transport both the metal nutrient iron (Fe) and the toxic metal cadmium (Cd) (Thomine et al., 2000; Cailliatte et al., 2009). However, their role in other abiotic stresses and phytohormones has not been elucidated till date. Interestingly, *NRAMP6* was found to be highly up-regulated in one of our SN13-induced salt stress transcript profiling study of rice seedlings (Unpublished). Accordingly, in this study this gene was found to be differentially expressed under all stresses with maximum expression at 3 h. It indicates possible *NRAMP*-regulated stress alleviation in rice. Our results and previous evidences regarding stress alleviation by PGPR suggest the crucial role of SN13 in positively modulating gene expression under various stresses and also indicate a possible gene-regulated cross-talk among all stresses and phytohormones treatments.

## Conclusion and Future Perspectives

**FIGURE 6:**
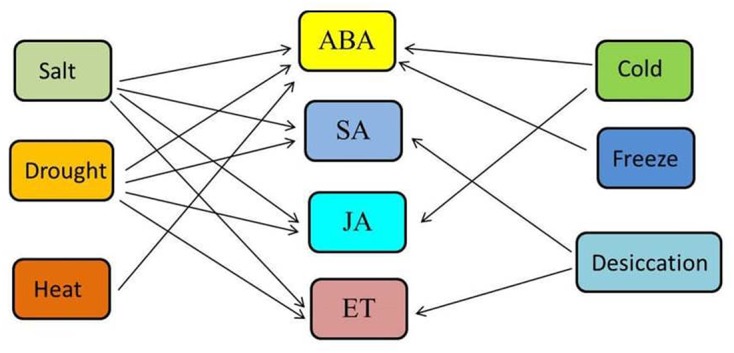
A model of the SN13-responsive cross-talk among various abiotic stresses and phytohormones operating in rice is created based on biochemical analyses and gene expression profiling. The phytohormones and abiotic stress interactions shown here is only a fraction of stress signal cross-talk occurring in rice when exposed to SN13 and different other cross-talk points are yet to be discovered.

This study highlights a beneficial bipartite plant–microbe interaction between rice seedlings and *B. amyloliquefaciens* SN13 under short-term abiotic stresses and phytohormone treatments. Taken together our results indicate that the abiotic stress amelioration capacity of rice seedlings have been significantly improved with SN13-inoculation under all stresses. Stress-induced symptoms in rice such as membrane integrity, accumulation of osmoprotectants, and expression of marker genes were significantly improved in presence of SN13. However, a more detailed study on the role of SN13 in improving stress tolerance of rice at subsequent developmental stages can be an interesting topic for further investigation. Based on differential responses of rice seedlings to abiotic stresses and phytohormones, PCA analysis confirmed basis for a holistic view on SN13 inoculation effects on rice response to different abiotic stresses and phytohormone treatments. This highlighted a possible PGPR-induced cross-talk among abiotic stresses and phytohormones (**Figure 6**). It can be deduced that SN13-responsive cross-talk is most extensive among all four phytohormones and salt and drought stresses as compared to heat, desiccation, cold, and freeze. Our results thus paves way for a more detailed understanding of various cross-talk points among phytohormones and stress signaling cascades in response to beneficial microbe(s) for their effective utilization in developing crop varieties with improved stress tolerance.

## Author Contributions

CL conceived and designed research. ST conducted experiments. ST, CL, VP, and PC analyzed data. CL and ST wrote the manuscript.

## Conflict of Interest Statement

The authors declare that the research was conducted in the absence of any commercial or financial relationships that could be construed as a potential conflict of interest.

## Supplementary material

FIGURE S1 Differential expression of *DHN* **(A)**, *GST* **(B)**, *LEA* **(C)**, *NAM* **(D)**, *GRAM* **(E)**, and *NRAMP6* **(F)** in rice exposed to salt, heat, ABA, and JA at 1, 3, 10, and 24 h in the presence or absence of SN13. Data represent the means ± SD of three independent experiments. Different letters on the graph indicate significant differences according to Duncan’s test (*P* ≤ 0.05).

FIGURE S2 Principal component analysis biplot of biochemical traits and gene expression of rice at 1 h **(A)**, 3 h **(B)**, 10 h **(C)** and 24 h **(D)** under abiotic stresses and phytohormone treatments in the presence or absence of SN13.
