# Supplementary figures and images for: Bacillus amyloliquefaciens Confers Tolerance to Various Abiotic Stresses and Modulates Plant Response to Phytohormones through Osmoprotection and Gene Expression Regulation in Rice
Source: Front Plant Sci. 2017 Aug 29;8:1510. doi: 10.3389/fpls.2017.01510 (PMC5581838; doi:10.3389/fpls.2017.01510)

Fig. S1

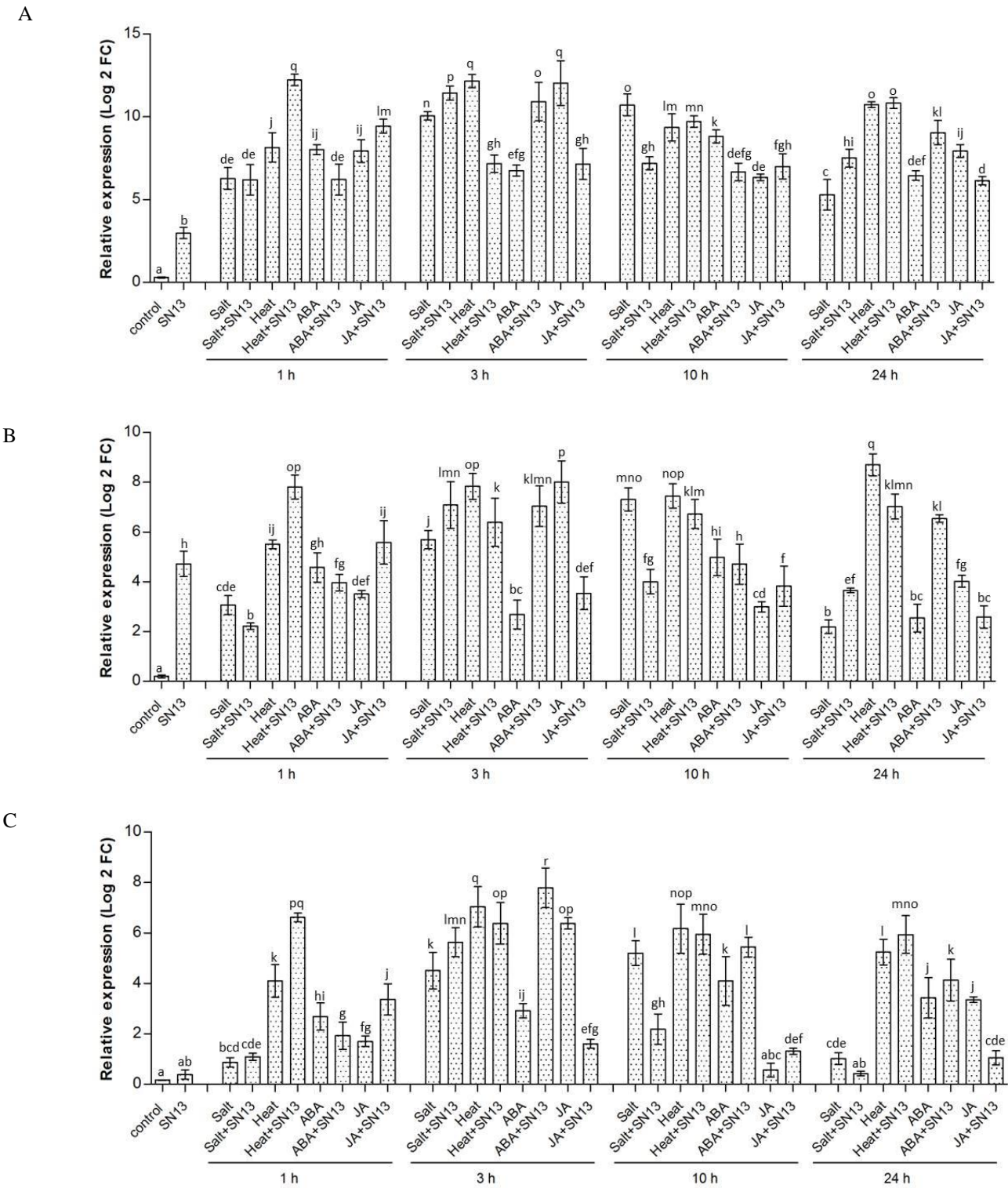

D

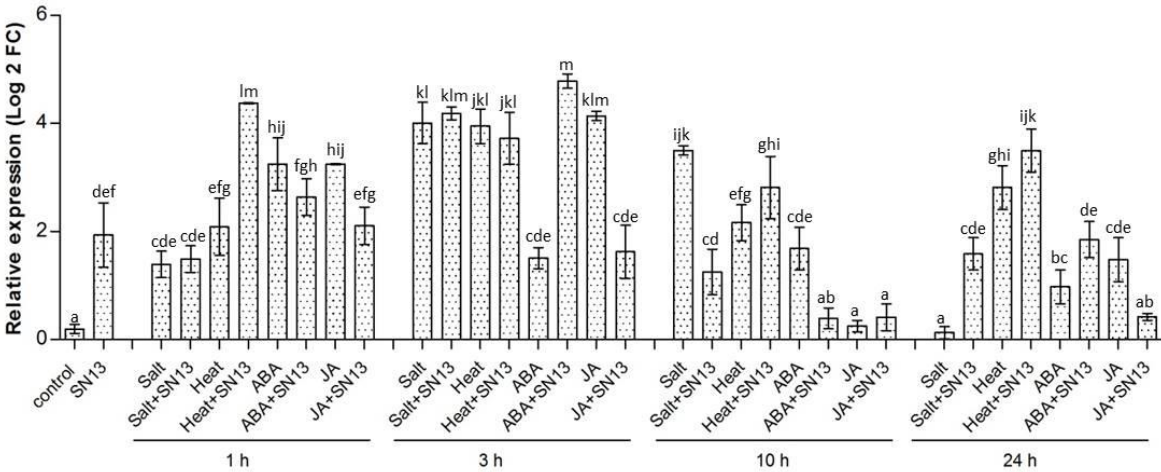

E

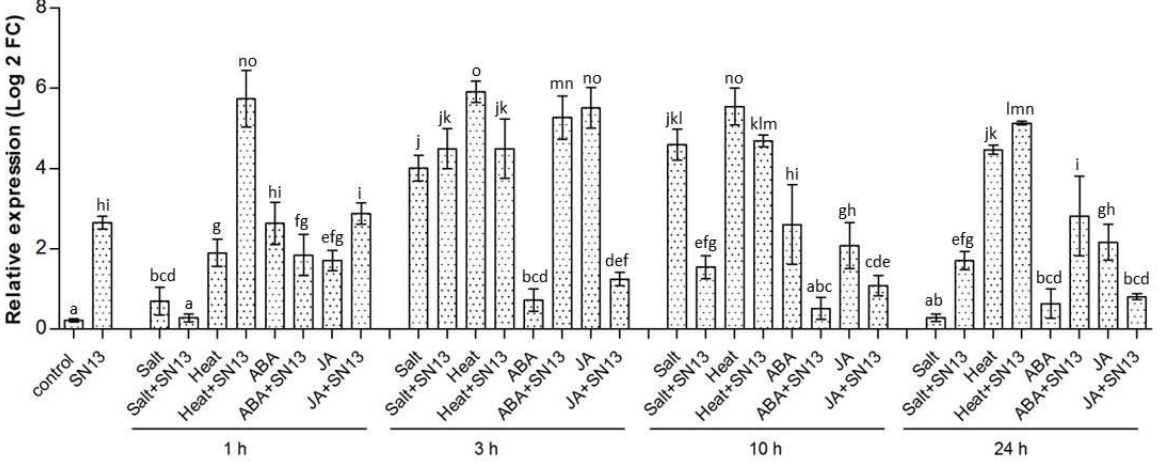

F

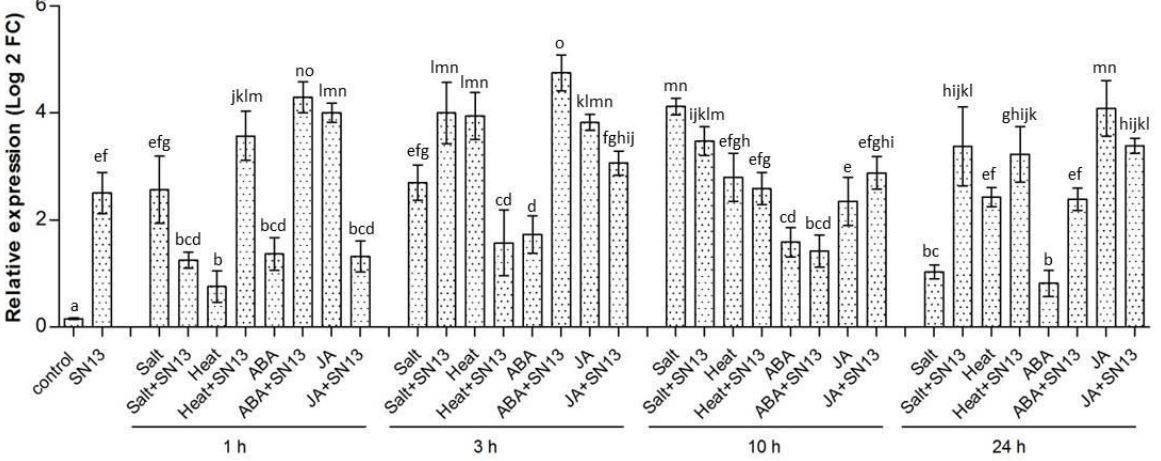

Supplement: FIGURE S1 — Differential expression of DHN (A), GST (B), LEA (C), NAM (D), GRAM (E), and NRAMP6 (F) in rice exposed to salt, heat, ABA, and JA at 1, 3, 10, and 24 h in the presence or absence of SN13. Data represent the means ± SD of three independent experiments. Different letters on the graph indicate significant differences according to Duncan’s test (P ≤ 0.05). [file Image_1.PDF]

Supplementary material Fig. S2

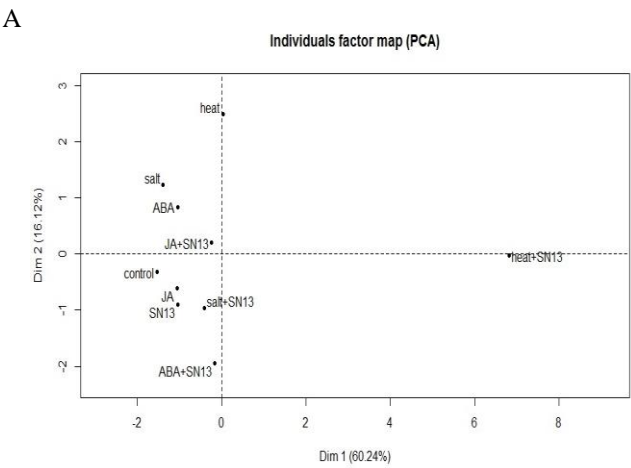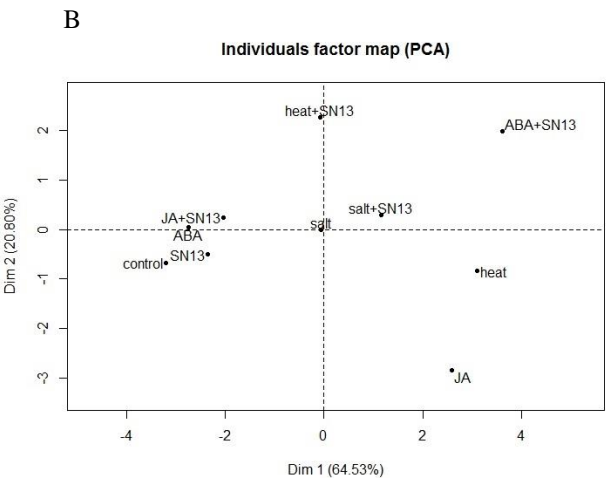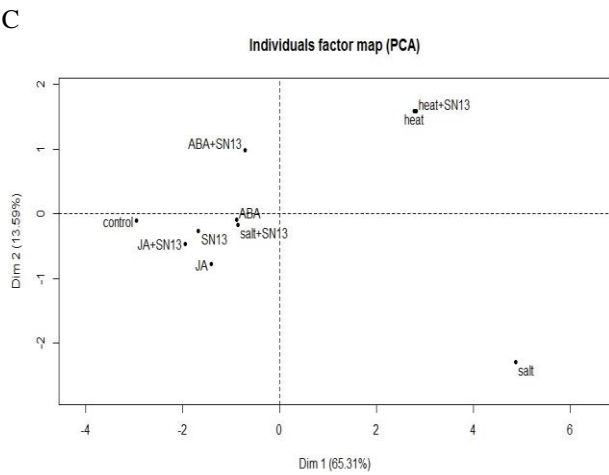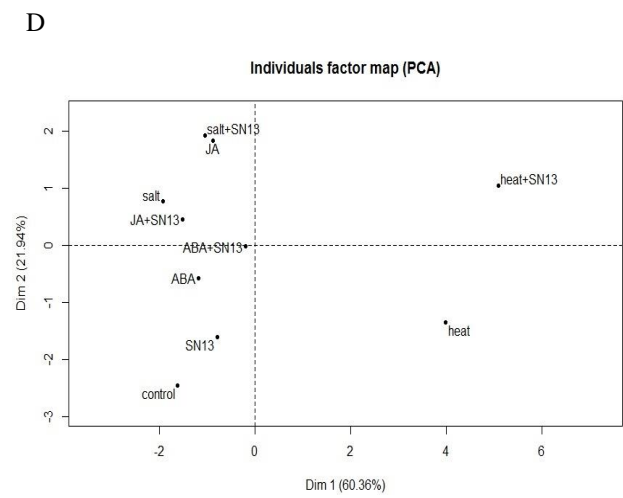

Supplement: FIGURE S2 — Principal component analysis biplot of biochemical traits and gene expression of rice at 1 h (A), 3 h (B), 10 h (C) and 24 h (D) under abiotic stresses and phytohormone treatments in the presence or absence of SN13. [file Image_2.PDF]
